# Functional MRI brain state occupancy in the presence of cerebral small vessel disease **—** a pre-registered replication analysis of the Hamburg City Health Study

**DOI:** 10.1162/imag_a_00122

**Published:** 2024-04-25

**Authors:** Thies Ingwersen, Carola Mayer, Marvin Petersen, Benedikt M. Frey, Jens Fiehler, Uta Hanning, Simone Kühn, Jürgen Gallinat, Raphael Twerenbold, Christian Gerloff, Bastian Cheng, Götz Thomalla, Eckhard Schlemm

**Affiliations:** Department of Neurology, University Medical Center Hamburg-Eppendorf, Hamburg, Germany; Department of Neuroradiology, University Medical Center Hamburg-Eppendorf, Hamburg, Germany; Department of Psychiatry, University Medical Center Hamburg-Eppendorf, Hamburg, Germany; Max-Planck-Institut für Bildungsforschung, Berlin, Germany; Department of Cardiology, University Medical Center Hamburg-Eppendorf, Hamburg, Germany

**Keywords:** brain states, cerebral small vessel disease, cognition, resting-state MRI, spatial coactivation pattern

## Abstract

We aimed to replicate recent findings on the association between the extent of cerebral small vessel disease (cSVD), functional brain network dedifferentiation, and cognitive impairment. We analyzed demographic, imaging, and behavioral data from the prospective population-based Hamburg City Health Study. Using a fully prespecified analysis pipeline, we estimated discrete brain states from structural and resting-state functional magnetic resonance imaging (MRI). In a multiverse analysis, we varied brain parcellations and functional MRI confound regression strategies. The severity of cSVD was operationalized as the volume of white matter hyperintensities of presumed vascular origin. Processing speed and executive dysfunction were quantified using the Trail Making Test (TMT). We hypothesized a) that a greater volume of supratentorial white matter hyperintensities would be associated with less time spent in functional MRI-derived brain states of high fractional occupancy; and b) that less time spent in these high-occupancy brain states associated with a longer time to completion in part B of the TMT. High-occupancy brain states were characterized by activation or suppression of the default mode network. Every5.1-fold increase in WMH volume was associated with a0.94-fold reduction in the odds of occupying DMN-related brain states (P =$5.01\times 10^{-8}$). Every5%increase in time spent in high-occupancy brain states was associated with a0.98-fold reduction in the TMT-B completion time (P =0.0116). Findings were robust across most brain parcellations and confound regression strategies. In conclusion, we successfully replicated previous findings on the association between cSVD, functional brain occupancy, and cognition in an independent sample. The data provide further evidence for a functional network dedifferentiation hypothesis of cSVD-related cognitive impairment. Further research is required to elucidate the mechanisms underlying these associations.

## 1 Introduction

Cerebral small vessel disease (cSVD) is an arteriolopathy of the brain associated with age and common cardiovascular risk factors (Wardlaw, C. Smith, & Dichgans, 2013). cSVD predisposes patients to ischemic stroke (in particular lacunar stroke) and may lead to cognitive impairment and dementia (Cannistraro et al., 2019). Neuroimaging findings in cSVD reflect its underlying pathology (Wardlaw et al., 2015) and include white matter hyperintensities (WMH), lacunes of presumed vascular origin, small subcortical infarcts and microbleeds, enlarged perivascular spaces, as well as brain atrophy (Wardlaw, E. E. Smith, Biessels, et al., 2013). However, the extent of visible cSVD features on magnetic resonance imaging (MRI) is an imperfect predictor of the severity of clinical sequelae (Das et al., 2019) and our understanding of the causal mechanisms linking cSVD-associated brain damage to clinical deficits remains limited (Bos et al., 2018).

Recent efforts have focused on exploiting network aspects of the structural (Tuladhar, et al., 2016,^2020^;Lawrence, Zeestraten, et al., 2018) and functional (Dey et al., 2016;Schulz et al., 2021) organization of the brain to understand the relationship between cSVD and clinical deficits in cognition and other domains that rely on distributed processing. Reduced structural network efficiency has repeatedly been described as a causal factor in the development of cognitive impairment, particularly executive dysfunction and reduced processing speed in cSVD (Lawrence, Chung, et al., 2014;Shen et al., 2020;Reijmer et al., 2016;Prins et al., 2005). Findings with respect to functional connectivity (FC), however, are more heterogeneous than their SC counterparts, perhaps because FC measurements are prone to be affected by hemodynamic factors and noise, resulting in relatively low reliability, especially with resting-state scans of short duration (Laumann et al., 2015). This problem is exacerbated in the presence of cSVD and worsened by arbitrary processing choices (Gesierich et al., 2020;Lawrence, Tozer, et al., 2018).

As a promising new avenue, time-varying, or dynamic, functional connectivity approaches have recently been explored in patients with subcortical ischemic vascular disease (Xu et al., 2021;Yin et al., 2022). Although the study of dynamic FC measures may not solve the problem of limited reliability, especially in small populations or participants with extensive structural brain changes, it adds another—temporal—dimension to the study of functional brain organization, which is otherwise overlooked. Importantly, FC dynamics not only reflect moment-to-moment fluctuations in cognitive processes, but are also related to brain plasticity and homeostasis (Laumann & Snyder, 2021;Laumann, et al., 2017), which may be impaired in cSVD.

In the present paper, we aimed to replicate and extend the main results ofSchlemm et al. (2022). In this recent study, the authors analyzed MR imaging and clinical data from the prospective Hamburg City Health Study (HCHS,Jagodzinski et al., 2020) using a coactivation pattern approach to define discrete brain states, and found associations between the WMH load, time spent in high-occupancy brain states characterized by activation or suppression of the default mode network (DMN), and cognitive impairment. Specifically, every 4.7-fold increase in WMH volume was associated with a 0.95-fold reduction in the odds of occupying a DMN-related brain state; every 2.5 seconds (i.e., one repetition time) not spent in one of those states was associated with a 1.06-fold increase in TMT-B completion times.

The fractional occupancy of a functional MRI-derived discrete brain state is a participant-specific measure of brain dynamics and is defined as the proportion of BOLD volumes assigned to that state relative to all BOLD volumes acquired during a resting-state scan.

Our primary hypothesis for the present work was that the volume of supratentorial white matter hyperintensities is associated with fractional occupancy of DMN-related brain states in a middle-aged to elderly population mildly affected by cSVD. Our secondary hypothesis was that fractional occupancy is associated with executive dysfunction and reduced processing speed, measured as the time to complete part B of the Trail Making Test (TMT).

Both hypotheses were tested in an independent subsample of the HCHS study population using the same imaging protocols, examination procedures, and analysis pipelines as those inSchlemm et al. (2022). The robustness of the associations was explored using a multiverse approach by varying key steps in the analysis pipeline.

## 2 Methods

### 2.1 Study population

This study analyzed data from the Hamburg City Health Study (HCHS), an ongoing prospective, population-based cohort study aiming to recruit a cross-sectional sample of45000adult participants from the city of Hamburg, Germany (Jagodzinski et al., 2020). From the first10000participants of the HCHS, we planned to include those who were documented to have received brain imaging (n = 2648) and exclude those who were analyzed in our previous report (Schlemm et al., 2022) (n = 970). The ethical review board of the Landesärztekammer Hamburg (State of Hamburg Chamber of Medical Practitioners) approved the HCHS (PV5131), and all participants provided written informed consent.

### 2.2 Demographic and clinical characterization

From the study database, we extracted the participants’ age at the time of inclusion in years, their sex, and the number of years spent in education. During the visit to the study center, participants underwent cognitive assessment using standardized tests. From the database, we extracted their performance scores on the Trail Making Test part B, measured in seconds, as an operationalization of executive function and psychomotor processing speed (Arbuthnott & Frank, 2000;Tombaugh, 2004). For descriptive purposes, we also extracted data on past medical history and reported the proportion of participants with a previous diagnosis of dementia.

### 2.3 MRI acquisition and preprocessing

The magnetic resonance imaging protocol for the HCHS includes structural and resting-state functional sequences. The acquisition parameters for a3 TSiemens Skyra MRI scanner (Siemens, Erlangen, Germany) have been previously reported (Frey et al., 2021;Petersen et al., 2020) and are given as follows:

For$T_{1}$-weighted anatomical images, a 3D rapid acquisition gradient-echo sequence (MPRAGE) was used with the following sequence parameters: repetition timeTR=2500ms, echo timeTE=2.12ms, 256 axial slices, slice thicknessST=0.94mm, and in-plane resolution$\text{IPR}=\left(0.83\times 0.83\right){\text{mm}}^{2}$.

$T_{2}$-weighted fluid attenuated inversion recovery (FLAIR) images were acquired with the following sequence parameters:TR=4700ms,TE=392ms,192axial slices,ST=0.9mm,$\text{IPR}=\left(0.75\times 0.75\right){\text{mm}}^{2}$.

125resting state functional MRI volumes were acquired (TR=2500ms;TE=25ms;flip angle=90°; slices =49;ST=3mm;slice gap=0mm;$\text{IPR}=\left(2.66\times 2.66\right){\text{ mm}}^{2}$). The participants were asked to keep their eyes open and to think of nothing.

We verified the presence and voxel dimensions of expected MRI data for each participant and excluded those for whom at least one of$T_{1}$-weighted, FLAIR, and resting-state MRI was missing. We also excluded participants with neuroradiologically confirmed space-occupying intra-axial lesion. To ensure reproducibility, no visual quality assessment of raw images was performed.

For the remaining participants, structural and resting-state functional MRI data were preprocessed using FreeSurfer v6.0 (https://surfer.nmr.mgh.harvard.edu/), and fMRIPrep v20.2.6 (Esteban et al., 2019), using default parameters. Participants were excluded if automated processing using at least one of these packages failed.

### 2.4 Quantification of WMH load

For our primary analysis, the extent of ischemic white matter disease was operationalized as the total volume of supratentorial WMH obtained from automated segmentation using a combination of anatomical priors, BIANCA (Griffanti et al., 2016), and LOCATE (Sundaresan et al., 2019), post-processed with a minimum cluster size of30voxels, as described inSchlemm et al. (2022). In an exploratory analysis, we partitioned voxels identified as WMH into deep and periventricular components according to their distance to the ventricular system (cut-off10mm,Griffanti et al., 2018).

### 2.5 Brain state estimation

The output from fMRIprep was post-processed using xcpEngine v1.2.3 to obtain de-confounded spatially averaged BOLD time series (Ciric et al., 2017). For the primary analysis, we used the*36p*regression strategy and the Schaefer-400parcellation (Schaefer et al., 2018), as inSchlemm et al. (2022).

Different atlases and confound regression strategies, as implemented in xcpEngine, were included in an exploratory multiverse analysis.

Co-activation pattern (CAP) analysis was performed by first aggregating parcellated, de-confounded BOLD signals into a$\left(n_{\text{parcels}}\times \sum_{i}n_{\text{time points},i}\right)$feature matrix, where$n_{\text{time points},i}$denotes the number of retained volumes for participantiafter confound regression. Clustering was performed using thek-means algorithm (k=5) with a distance measure given by 1 minus the sample Pearson correlation between points, as implemented in Matlab R2021a. We estimated the participant- and state-specific fractional occupancies, which are defined as the proportion of BOLD volumes assigned to each brain state (Vidaurre et al., 2018). The two states with the highest average occupancies were identified as the basis for further analysis.

### 2.6 Statistical analysis

For demographic (age, sex, and years of education) and clinical (TMT-B) variables, the number of missing items is reported. For non-missing values, we provide descriptive summary statistics using median and interquartile range. The proportions of men and women in the sample are reported. Since we expected based on our pilot data (Schlemm et al., 2022) that the proportion of missing data would be small, primary regression modelling was carried out as a complete-case analysis.

As an outcome-neutral quality check of the implementation of the MRI processing pipeline, brain state estimation, and co-activation pattern analysis, we compared fractional occupancies between brain states. We expected that the average fractional occupancy in the two high-occupancy states would be higher than the average fractional occupancy in the other three states. Point estimates and 95% confidence intervals are presented for the difference in average fractional occupancy to verify this assertion.

For further analyses, non-zero WMH volumes were subjected to logarithmic transformation. Zero values retained their value of zero; to compensate, all models included a binary indicator for zero WMH volume if at least one non-zero WMH value was present.

To assess the primary hypothesis of a negative association between the extent of ischemic white matter disease and time spent in high-occupancy brain states, we performed a fixed-dispersion Beta regression to model the logit of the conditional expectation of the average fractional occupancy of two high-occupancy states as an affine function of the logarithmized WMH load. Age and sex were included as covariates. The strength of the association was quantified as the odds ratio per interquartile ratio of the WMH burden distribution, and is accompanied by a 95% confidence interval. Significance testing of the null hypothesis of no association was conducted at the conventional significance level of 0.05. Estimation and testing were carried out using the “betareg” package v3.1.4 in R v4.2.1.

To assess the secondary hypothesis of an association between time spent in high-occupancy brain states and executive dysfunction, we performed a generalized linear regression with a Gamma response distribution to model the logarithm of the conditional expected completion time in part B of the TMT as an affine function of the average fractional occupancy of two high-occupancy states. Age, sex, years of education, and logarithmized WMH load were included as covariates. The strength of the association was quantified as a multiplicative factor per percentage point and accompanied by a 95% confidence interval. Significance testing of the null hypothesis of no association was conducted at the conventional significance level of 0.05. Estimation and testing were performed using the glm function included in the “stats” package from R v4.2.1.

### 2.7 Pre-registered analyses

The analysis plan was pre-registered on June 27, 2023 athttps://osf.io/fcqmb. The sample size calculation was based on an effect size on the odds ratio scale of 0.95, corresponding to an absolute difference in the probability of occupying a DMN-related brain state between the first and third WMH-load quartile of 1.3 percentage points, and between the 5% and 95% percentile of 3.1 percentage points. Approximating half the difference in fractional occupancy of DMN-related states between different task demands (rest vs n-back) in healthy participants, which was estimated to lie between 6 and 7 percentage points (Cornblath et al., 2020), this value represented a plausible choice for the smallest effect size of theoretical and practical interest. It also equals the estimated effect size based on the data presented inSchlemm et al. (2022).

Simple bootstrapping was used to create10000hypothetical datasets of size200,400,600,800,900,910,… ,1090,1100,1200,1400,1500, and1600. Each dataset was then subjected to the estimation procedure described above. For each sample size, the proportion of datasets in which the primary null hypothesis of no association between fractional occupancy and WMH load could be rejected atα=0.05was computed and recorded as a power curve inFigure 1.

**Fig. 1.**
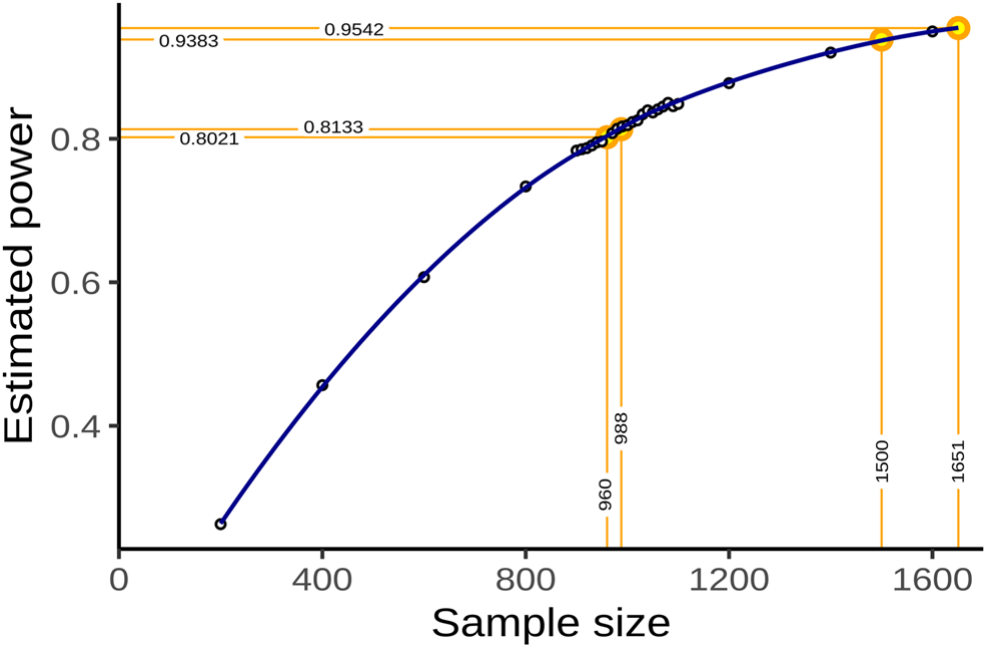
Sample size and power estimation. A-priori estimated power for different sample sizes was obtained as the proportion of synthetic data sets in which the null hypothesis of no association between WMH volume and time spent in high-occupancy brain states could be rejected at theα=0.05significance level. Proportions are based on a total of10000synthetic data sets obtained by bootstrapping the data presented inSchlemm et al. (2022). Highlighted in orange are the smallest sample size ensuring a power of at least80%(n=960), the sample size of the pilot data (n=988, post-hoc power81.3%), the expected sample sample size for this replication study (n=1500, a-priori power93.8%), and the achieved sample size (n=1651, a-priori power95.4%).

A sample size of960would have allowed the replication of the reported effect with a power of80.2%. We had anticipated a sample size of1500, which would have yielded a power of93.8%.

### 2.8 Multiverse analysis

In both (Schlemm et al., 2022) and our primary replication analysis, we made certain analytical choices in the operationalization of brain states and ischemic white matter disease, namely the use of the*36p*confound regression strategy, the Schaefer-400parcellation, and a BIANCA/LOCATE-based WMH segmentation algorithm. The robustness of the association between WMH burden and time spent in high-occupancy states with regard to other choices was explored in a multiverse analysis (Steegen et al., 2016). Specifically, in an exploratory analysis, we estimated brain states from BOLD time series processed according to a variety of established confound regression strategies and aggregated over different cortical brain parcellations (Table 2,Ciric et al., 2017,^2018^). The extent of cSVD was additionally quantified by the volume of deep and periventricular white matter hyperintensities.

For each combination of analytical choice of confound regression strategy, parcellation, and subdivision of white matter lesion load (9×9×3=243scenarios in total), we quantified the association between WMH load and average time spent in high-occupancy brain states using odds ratios and95%confidence intervals as described above.

No hypothesis testing was performed for these multiverse analyses. Rather, they serve to inform about the robustness of the outcome of the test of the primary hypothesis. Any substantial conclusions about the association between the severity of cerebral small vessel pathology and the time spent in high-occupancy brain states were drawn from the primary analysis using pre-specified methodological choices, as stated in the Scientific Question inTable 1.

**Table 1.** Study Design.

Question	Hypothesis	Sampling plan	Analysis plan	Rationale for deciding the sensitivity of the test	Interpretation given different outcomes	Theory that could be shown wrong by the outcome
Is severity of cerebral small disease, quantified by the volume of supratentorial white matter hyperintensities of presumed vascular origin (WMH), associated with time spent in high-occupancy brain states, defined by resting-state functional MRI?	( **Primary** ) Higher WMH volume is associated with lower average occupancy of the two highest-occupancy brain states.	Available participants with clinical and imaging data from the the HCHS ( Jagodzinski et al., 2020 )	Standardized preprocessing of structural and functional MRI data • automatic quantification of WMH • co-activation pattern analysis • multivariable generalized regression analyses	Tradition	P<0.05 —> rejection of the null hypothesis of no association between cSVD and fractional occupancy; P>0.05 —> insufficient evidence to reject the null hypothesis	Functional brain dynamics are not related to subcortical ischemic vascular disease.
Is time spent in high-occupancy brain states associated with cognitive impairment, measured as the time to complete part B of the trail making test (TMT)?	( **Secondary** ) Lower average occupancy of the two highest-occupancy brain states is associated with longer TMT-B time.	as above	as above	as above	P<0.05 —> rejection of the null hypothesis of no association between fractional occupancy and cognitive impairment; P>0.05 —> insufficient evidence to reject the null hypothesis	Cognitive function is not related to MRI-derived functional brain dynamics.

Overview of the Scientific Questions addressed in the present study (first column), the two main hypotheses being investigated (second column), and details of the underlying study.

**Table 2.** Multiverse analysis.

Name of the atlas	#parcels	Reference
Desikan–Killiany	86	Desikan et al., 2006
AAL	116	Tzourio-Mazoyer et al., 2002
Harvard–Oxford	112	Makris et al., 2006
glasser360	360	Glasser et al., 2016
gordon333	333	Gordon et al., 2016
power264	264	Power et al., 2011
schaefer{N}	100	Schaefer et al., 2018
	200	
	400	
**(a)** Parcellations		
Design	Reference	
24p	Friston et al., 1996	
24p + GSR	Macey et al., 2004	
36p	Satterthwaite et al., 2013	
36p + spike regression	Cox, 1996	
36p + despiking	Satterthwaite et al., 2013	
36p + scrubbing	Power et al., 2014	
ACompCor	Muschelli et al., 2014	
TCompCor	Behzadi et al., 2007	
AROMA	Pruim et al., 2015	
**(b)** Confound regression strategies, adapted from ( Ciric et al., 2017 )		

Overview over different brain parcellations and confound regression strategies implemented using xcpEngine (Ciric et al., 2018). A total of9×9=81analytical combinations were explored to assess the robustness of our results with respect to these processing choices.

AAL: Automatic Anatomical Labeling; AROMA: Automatic Removal of Motion Artifacts; GSR: Global signal regression.

### 2.9 Further exploratory analysis

In previous work, two high-occupancy brain states have been related to the default mode network (Cornblath et al., 2020). We further explored this relationship by computing, for each individual brain state, the cosine similarity of the positive and negative activations of the cluster’s centroid with a set of a priori defined functional ‘communities’ or networks (Schaefer et al., 2018;Yeo et al., 2011). The results were visualized as spider plots for the Schaefer atlases.

In further exploratory analyses, we describe the associations between brain state dynamics and other measures of cognitive ability such as memory and language.

### 2.10 Pilot data and analysis

Summary data from the first1000imaging data points of the HCHS have been published withSchlemm et al. (2022)and formed the basis for the hypotheses tested in this replication study. Before preregistration, we had implemented our prespecified analysis pipeline described above in R and Matlab, and applied it to this previous sample. Data, code, and results from this pilot analysis have been stored with the archived Stage 1 report on GitHub (https://github.com/csi-hamburg/HCHS-brain-states-RR, v1.5) and preserved on Zenodo.

### 2.11 Timeline and access to data

At the time of planning of this study, all demographic, clinical, and imaging data used in this analysis had been collected by the HCHS and were held in the central trial database. Quality checks for non-imaging variables had been performed centrally. WMH segmentation based on structural MRI data of the first10000participants of the HCHS had been performed previously using the BIANCA/LOCATE approach (Rimmele et al., 2022). Functional MRI data and clinical measures of executive dysfunction (TMT-B scores) had not previously been analyzed by the pre-registering author (ES).

### 2.12 Deviations from preregistration

For deconfounding and aggregating BOLD data at brain parcellation level, the software xcpEngine was used in version 1.2.3, not 1.2.1, to ensure that that the correct MNI reference template (MNI152NLin2009cAsym) is used for registration of brain atlases. This decision was made before analyzing the data.

## 3 Results

For this replication study, a total of2648datasets were available, of which970were already included in our previous analysis and thus discarded. In13of the resulting1678datasets, one or more MRI sequences were missing. Of the complete datasets (n =1665), we excluded5participants due to intra-axial space-occupying lesions. An additional9participants were excluded because of unsuccessful preprocessing, WMH segmentation, or xcpEngine failure, resulting in1651datasets for analysis. A study-flowchart is provided inFigure 2.

**Fig. 2.**
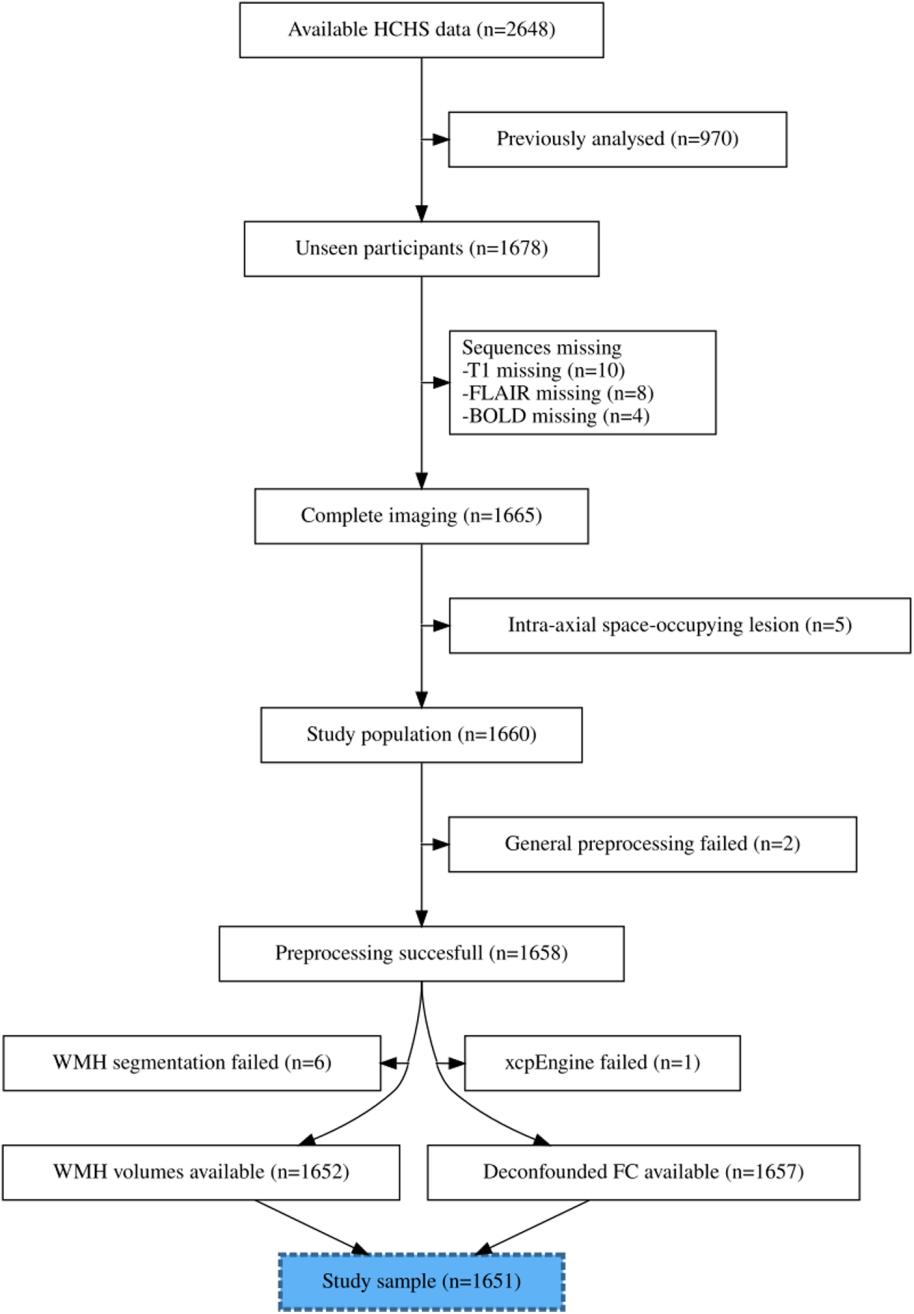
Study flowchart. Composition of the study population after application of inclusion and exclusion criteria, and image processing.

Baseline demographic and cognitive values, including the number of missing items, are reported inTable 3.

**Table 3.** Descriptive statistics of the study population.

	N = 1651
*Demographics* (no Missing, n (%))	
Age, yr	
Median (IQR)	66 (59–72)
Sex	
Male	940/1651 (57%)
Female	711/1651 (43%)
*Cardiovascular risk factors*	
Hypertension	
Present	1177/1611 (73.1%)
Missing n (%)	40 (2.4%)
Diabetes	
Present	157/1566 (10%)
Missing n (%)	85 (5.1%)
Smoking	
Present	200/1360 (14.7%)
Missing n (%)	201 (12.9%%)
Hyperlipidaemia	
Present	426/1578 (27%)
Missing n (%)	73 (4.4%)
*Cognitive test results*	
MMSE, # (max. 30)	
Median (IQR)	28 (27–29)
Missing n (%)	129 (7.8%)
Vocabulary (MWT-B), # (max. 37)	
Median (IQR)	32 (30–34)
Missing n (%)	295 (18%)
Word recall, # (max. 10)	
Median (IQR)	8 (6–9)
Missing n (%)	180 (11%)
Animal Naming	
Median (IQR)	24 (20–29)
Missing n (%)	116 (7.0%)
TMT-A, seconds	
Median (IQR)	38 (31–48)
Missing n (%)	144 (8.7%)
TMT-B, seconds	
Median (IQR)	83 (65–110)
Missing n (%)	162 (9.8%)
*History*	
Diagnosed dementia	
Present	6/1645 (0.4%)
Missing n (%)	6 (0.4%)
Years of education	
Median (IQR)	13 (12–16)
Missing n (%)	34 (2%)

Data are presented as median (interquartile range) or count (percentage) of non-missing items, as appropriate. Number of percentage of missing items is reported separately.

WMH volumes (median1.05mL, IQR0.47mLto2.37mL), motion estimates, and fractional occupancies of brain states 1 through 5 are reported inTable 4.

**Table 4.** Structural and functional imaging characteristics

	N = 1651
WMH volume ^1^ , mL	
Total	1.05 (0.47–2.37), 9 Z
Periventricular	0.94 (0.43–2.04), 9 Z
Deep	0.10 (0.03–0.37), 344 Z
Motion during rs-fMRI	
Framewise displacement, mm	0.21 (0.15–0.63)
RMSD, mm	0.086 (0.058–0.12)
DVARS	27.8 (24.3–31.8)
Fractional occupancy, %	
DMN+	24.8 (20.8–28.0)
DMN-	24.0 (20.0–28.0)
S3	18.4 (15.2–22.4)
S4	16.8 (12.8–20.8)
S5	15.2 (12.0–19.2)

Data are presented as median (interquartile range). Supratentorial WMH volumes were obtained by semiautomatic segmentation of FLAIR images using a BINACA/LOCATE-basedk-nearest neighbors algorithm and stratified by their distance to the lateral ventricles (<10mm, periventricular; >10mm, deep). Motion parameters were estimated during fMRIprep processing of BOLD scans. Fractional occupancies were calculated by assigning individual BOLD volumes to one of five discrete brain states defined by k-means clustering-based co-activation pattern analysis. Two high-occupancy states are labeled DMN+ and DMN, in view of their network connectivity profiles as shown inFigure 6.

1 Number of zero values indicated by Z.

In an outcome-neutral quality check of the implementation of (i) the MRI processing pipeline, (ii) brain state estimation, and (iii) co-activation pattern analysis, the mean difference in fractional occupancy between high- and low-occupancy states was consistently maintained, with a point-estimate of the separation between two high-occupancy and three low-occupancy states of6.7%(95%confidence interval,6.2%to7.1%) in the*36p*paradigm. This indicates that the implementation of the pipeline was correct and that the brain state estimation and co-activation pattern analysis worked as intended.

### 3.1 Pre-registered hypotheses

#### 3.1.1 Association between WMH load and fractional occupancy

The results of the test of our primary preregistered hypothesis of an association between supratentorial WMH volume and the time spent in high-occupancy brain states are shown inFigure 3andTable 5.

**Fig. 3.**
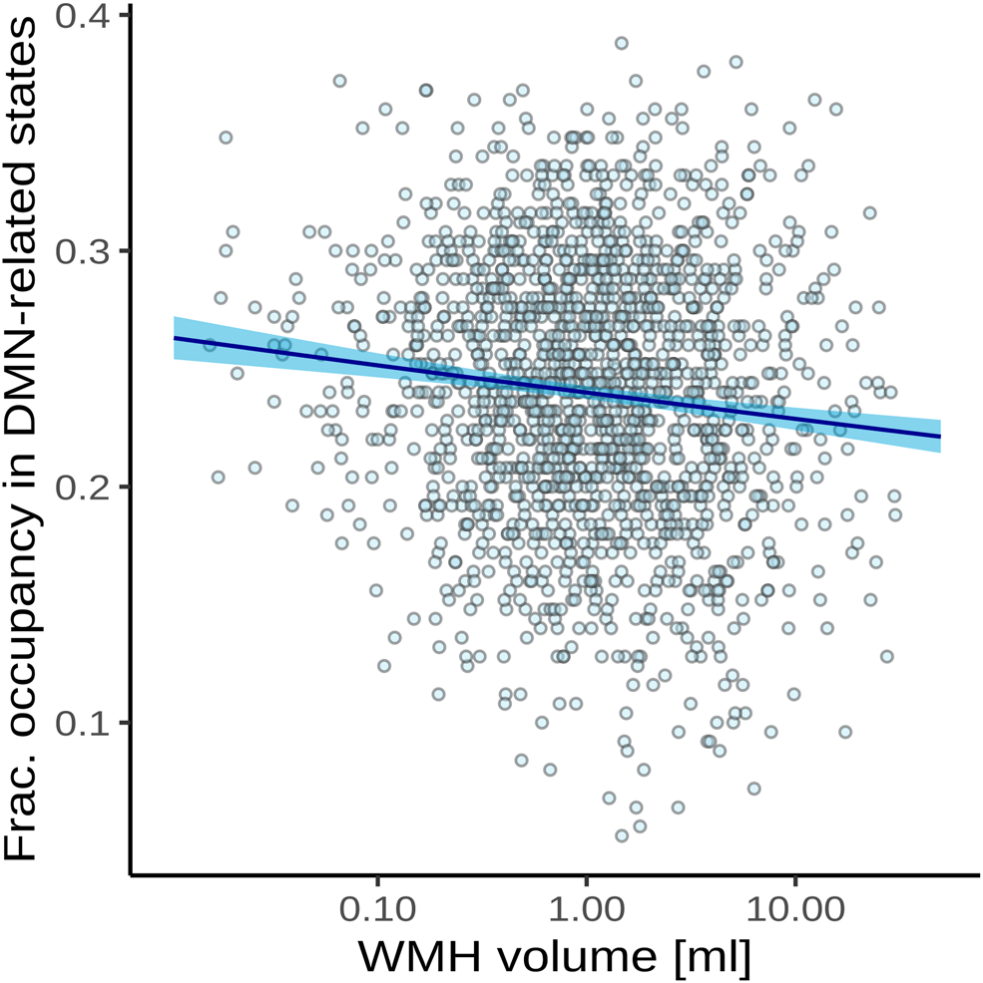
Association between time spent in high-occupancy brain states and supratentorial WMH volume. Point estimates (black line) and 95%-confidence region (light blue ribbon) of the conditional mean fractional occupancy are obtained from unadjusted beta regression modeling. Each marker represents one of N = 1642 independent participants with a non-zero total WMH volume.

**Table 5.** Association between time spent in high-occupancy DMN-related brain states and WMH volume adjusted for age and sex.

	Estimate	P	95%-CI
Intercept	0.24	<0.0001	0.21–0.27
WMH, per 5.1-fold increase ^1^	0.94	<0.0001	0.92–0.96
Age, per 10 years	1.04	0.001	1.01–1.06
Female sex	1.12	<0.0001	1.09–1.16
$1_{\left\{\text{WMH}=0\right\}}$	0.93	0.477	0.75–1.14

Beta regression table estimated fromn=1651independent participants using the model equation${\text{FO}}^{\text{high}}∼{\text{logWMH}}^{+}+1_{\left\{\text{WMH}=0\right\}}$+age+sex.

1 Interquartile ratio2.37/0.468=5.06.

Adjusted for age and sex, there was a0.94-fold reduction in the odds of occupying a high-occupancy brain state for every5.1-fold increase in WMH load (P =$5.01\times 10^{-8}$).

#### 3.1.2 Association between executive function and fractional occupancy in DMN-related states

The results of the test of our secondary preregistered hypothesis of an association between time spent in high-occupancy brain states and executive function as measured by the complete part B of the TMT are shown inFigure 4andTable 6.

**Fig. 4.**
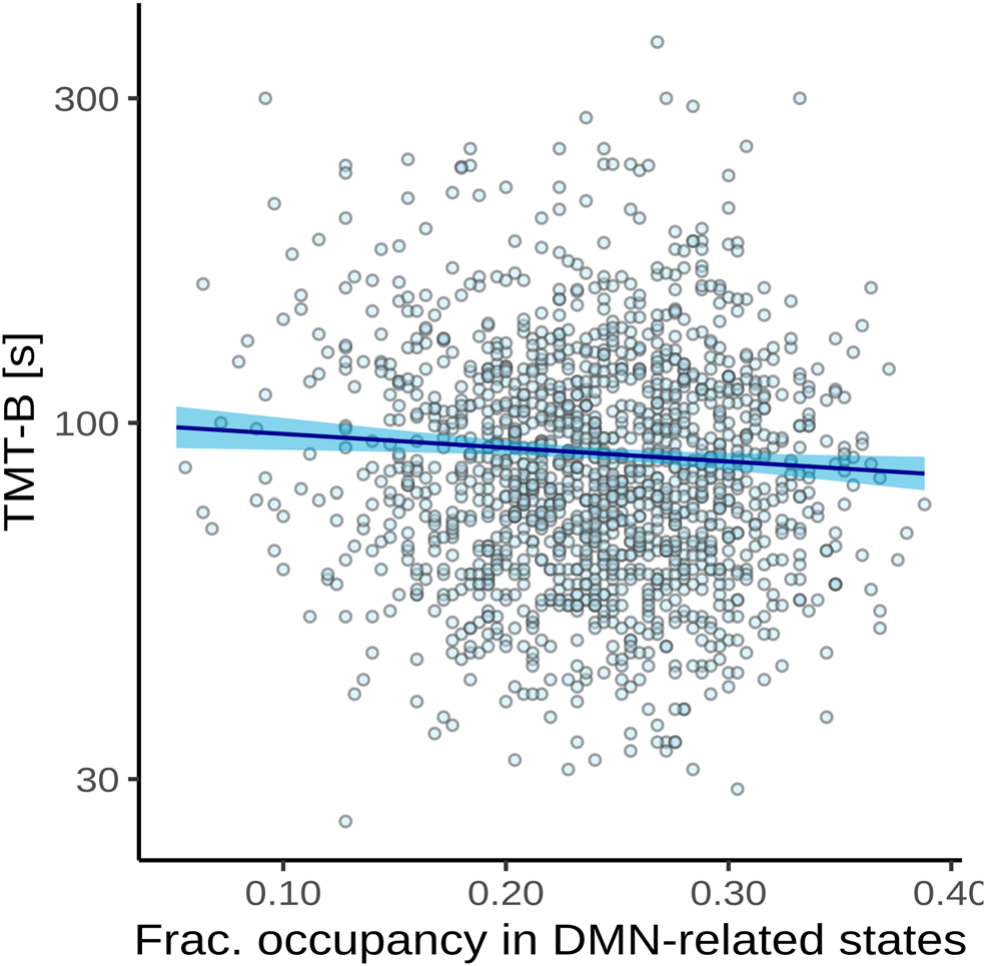
Association between time spent in high-occupancy DMN-related brain states and TMT-B completion time. Point estimates (black line) and 95%-confidence region (light blue ribbon) of the conditional mean TMT-B completion time are obtained from unadjusted Gamma regression modeling. Each marker represent one of N = 1482 independent participants with non-zero total WMH volume and available TMT-B data.

**Table 6.** Association between TMT-B and time spent in high-occupancy DMN-related brain states adjusted for age, sex, WMH volume, and years of education.

	Estimate	P	95% CI
Intercept	53.41	<0.0001	42.7–66.8
${\text{FO}}^{\text{high}}$ , per 5%	0.98	0.0116	0.96–0.99
WMH, per 5.1-fold increase ^1^	1.01	0.367	0.98–1.05
Age, per 10 years	1.18	<0.0001	1.15–1.21
Female sex	0.99	0.666	0.95–1.03
Education, per year	0.97	<0.0001	0.97–0.98
$1_{\left\{\text{WMH}=0\right\}}$	0.97	0.398	0.92–1.03

Gamma regression table estimated fromn=1483independent participants using the model equation$\text{TMT}-\text{B}∼{\text{FO}}^{\text{high}}+{\text{logWMH}}^{+}$$+1_{\left\{\text{WMH}=0\right\}}+\text{age}+\text{sex}+\text{educationyears}$.

1 Interquartile ratio2.37/0.468=5.06

Adjusted for age, sex, WMH volume, and years of education, there was a0.98-fold reduction in the time to complete the TMT-B for every5%increase in the time spent in high-occupancy brain states (P =0.0116).

### 3.2 Multiverse analysis

In a multiverse analysis, the main findings of associations between WMH load and FO and, to a lesser extent, between FO and TMT-B were robust with respect to the processing choices of brain parcellation and confound regression strategy.

**Fig. 5.**
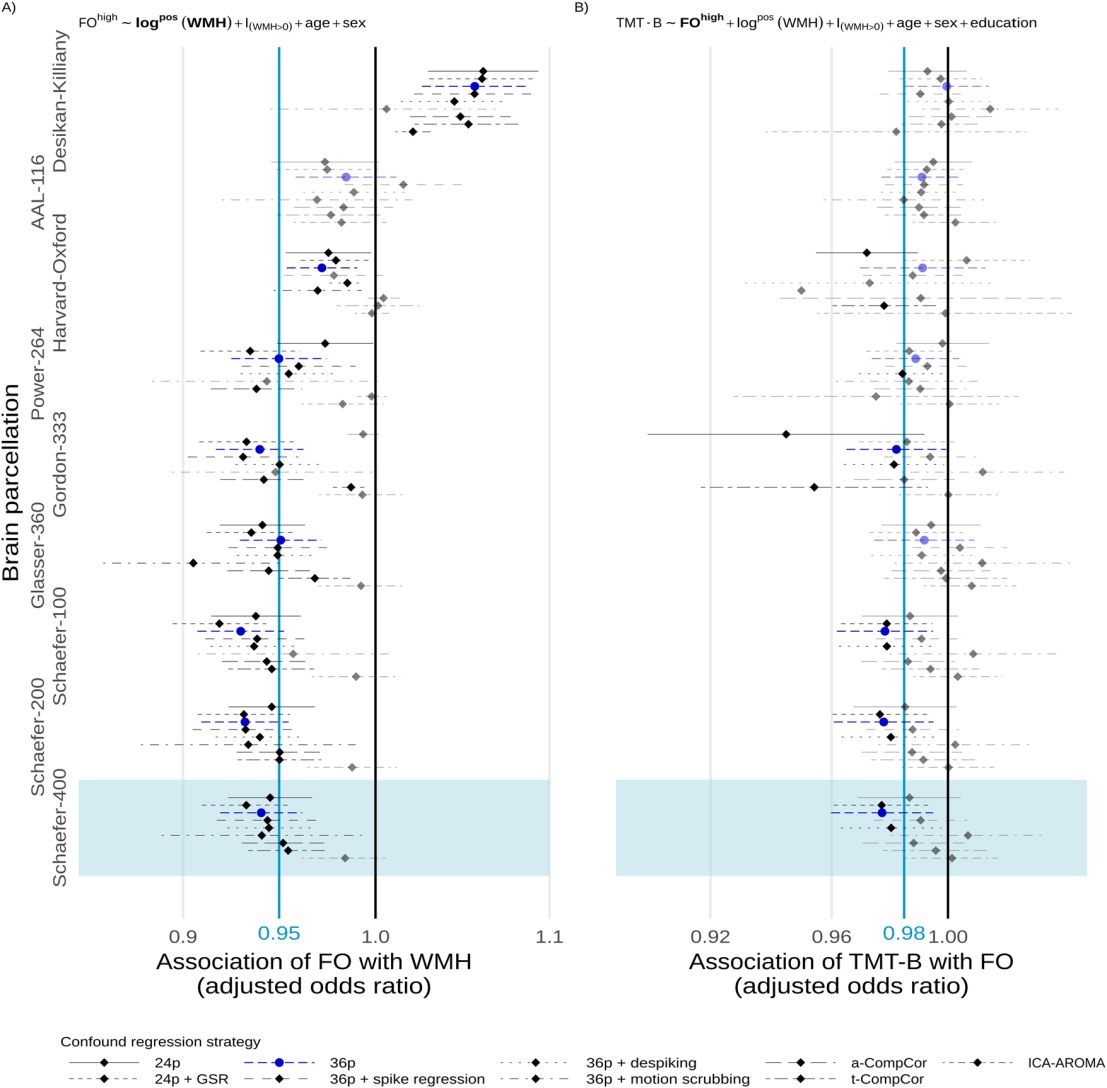
Multiverse analysis. Adjusted effect size estimates of the associations between cSVD severity (WMH volume) and network dedifferentiation (less time spent in high-occupancy DMN-related brain states) (A), and between network dedifferentiation and executive function (TMT-B completion time) (B). Effect sizes are given per 5.1-fold increase in WMH volume and a 5%-increase in fractional occupancy, respectively. Markers and line segments indicate point estimates and 95%-confidence intervals for adjusted odds ratios for different combinations of confound regression strategy and brain parcellation. The primary analytical choices are indicated by dark blue circles (36p) and light blue shading (Schaefer-400). Model equations for beta and gamma regressions, respectively, are given at the top. Vertical lines indicate no effect (black) and the effect size observed in the discovery cohort (Schlemm et al., 2022) (light blue), respectively, for reference. Effect sizes not reaching nominal statistical significance (α=0.05) are desaturated. Corresponding data based on periventricular and deep WMH volumes are presented in theAppendix.

**Appendix Fig. 1.**
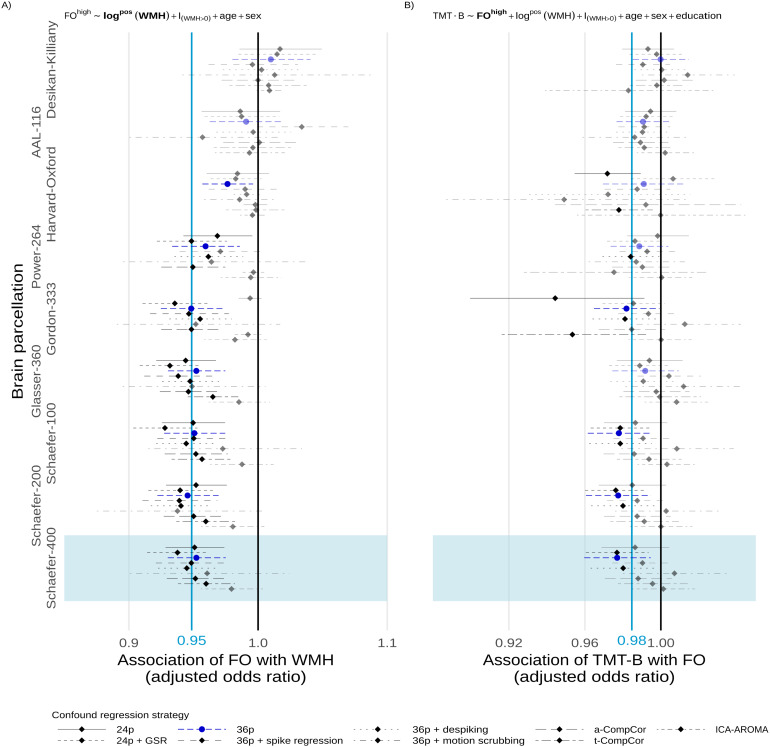
Multiverse analysis. Adjusted effect size estimates of the associations between cSVD severity (deep WMH volume) and network dedifferentiation (less time spent in high-occupancy DMN-related brain states) (A), and between network dedifferentiation and executive function (TMT-B completion time) (B). For details, see caption toFig. 5.

A nominally statistically significant negative association between the total WMH load and time spent in high-occupancy states was observed in 48 out of 81 scenarios, with 8 out of 81 significant positive associations occurring with the Desikan–Killiany parcellation only (Fig. 5A). For periventricular (deep) WMH volume, the results were similarly robust with 49 out of 81 (39/81) negative and 8 out of 81 (0/81) positive associations of nominal statistical significance, respectively (Appendix Figures 1and2).

The secondary finding of an association between greater TMT-B times and lower fractional occupancy was less robust with only 16 out of 81 nominally statistically significant negative and no significant positive associations, irrespective of operationalization of cSVD (total vs. periventricular vs. deep WMH volume) (Fig. 5B,Appendix Figures 1and2).

### 3.3 Additional analyses

#### 3.3.1 Connectivity profiles of brain states—relation to default mode network

Based on the cosine similarity between positive and negative activations of cluster centroids and indicator vectors of pre-defined large-scale brain networks, network activation profiles were computed for brain states estimated from Schaefer parcellations of varying spatial resolutions.

**Fig. 6.**
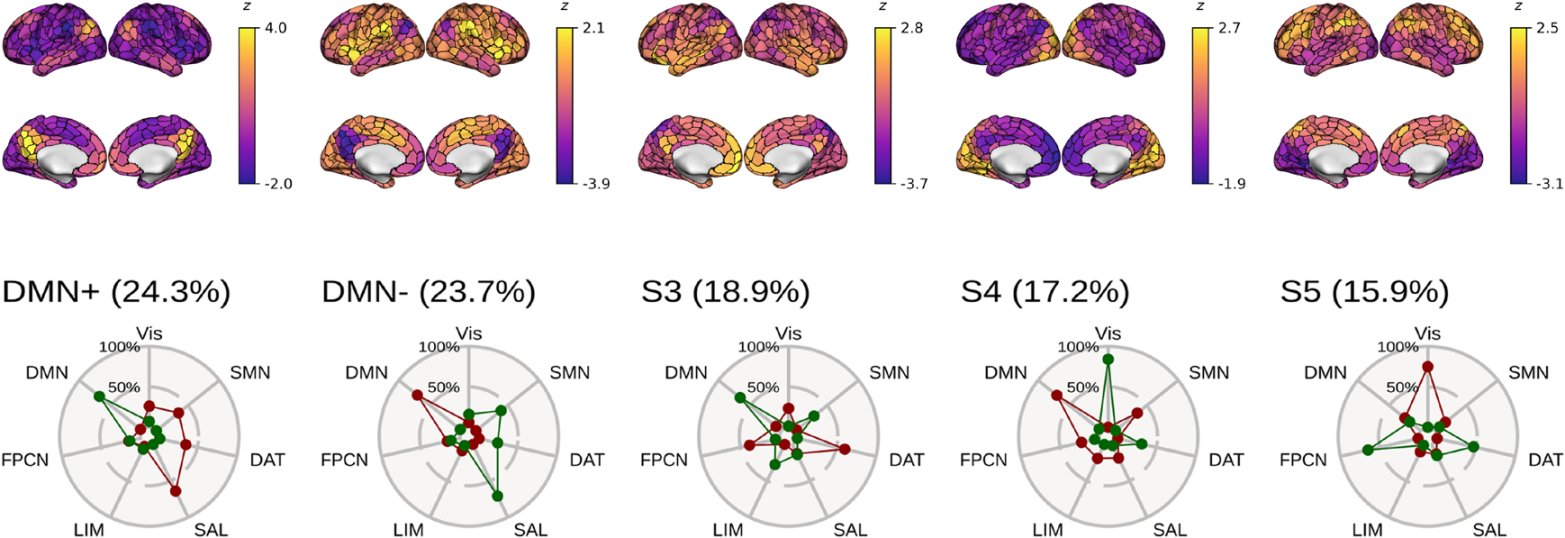
Connectivity profiles of brain states. [Top] Centroids of each identified brain state visualized in brain space. Note the individual color scales. [Bottom] Cosine similarity between centroids of brain states and signed indicator vectors corresponding to activation (green) and suppression (red) of each of seven predefined large-scale functional brain networks (Yeo et al., 2011). States are ordered by mean fractional occupancy across N = 1651 independent participants, indicated by parenthetical percentages. Two high-occupancy states are characterized by activation or suppression of the DMN; the remaining three low-occupancy states (S3–5) were not used in the present study. Note that mean FO values are similar, but not identical, to median FO values reported inTable 4.

Figure 6shows the corresponding spider plots, identifying states characterized by activation (DMN+) or suppression (DMN-) of the default mode network as states with the highest fractional occupancy.

#### 3.3.2 Association with other cognitive domains

Associations between the time spent in high-occupancy DMN-related brain states and cognitive measures beyond TMT-B are shown inFigure 7.

**Fig. 7.**
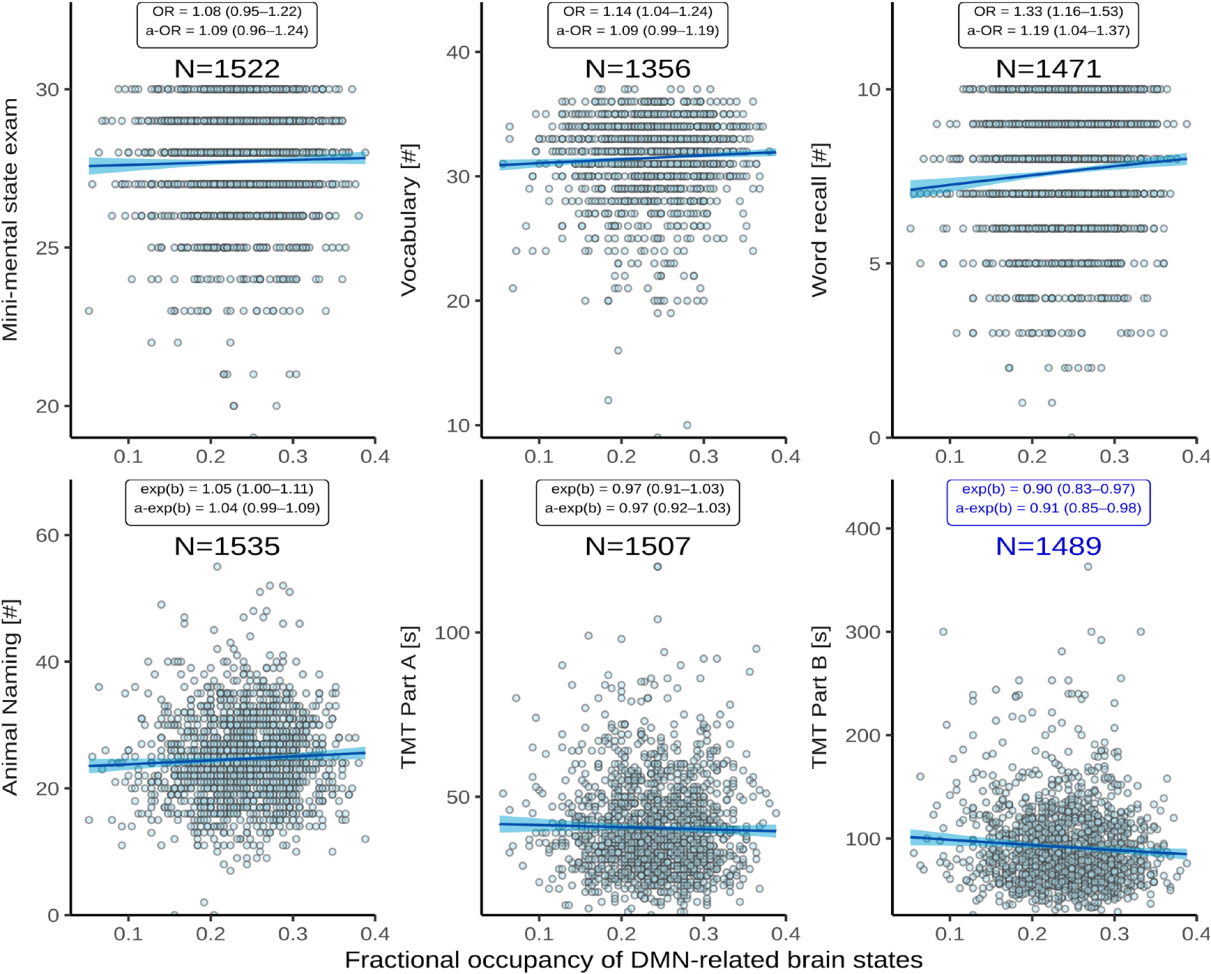
Association between time spent in high-occupancy DMN-related brain states and cognitive measures. Point estimates (black line) and 95%-confidence region (light blue ribbon) of the conditional mean cognitive measures are obtained from unadjusted binomial (top row: Mini-Mental State Examination, Vocabulary, Word List Recall, logit link) and Gamma regression (bottom row: Animal Naming, Trail Making Test [TMT] A/B: log link) modeling. Each marker represents one of N independent participants, as indicated. Insets report effect sizes with (adjusted [a-]) and without adjustment for the nuisance variables age, sex, WMH volume (coded as inFig. 5), and years of education. Effect sizes were quantified as odds ratios (ORs) (top) or response scale multipliers [exp(b)] (bottom) complemented by 95%-confidence intervals, and correspond to a 20%-increase in fractional occupancy. Note the different reference change in FO compared toTable 6chosen to adequately represent some of the smaller effect sizes. The bottom right panel highlighted in dark blue reproducesFigure 4.

Adjusted for age, sex, WMH load, and years of education, FO in DMN-related states appeared to be associated with better word recall (adjusted OR 1.19, nominal P 0.013), but not with global cognitive functioning (MMSE, adjusted OR 1.09) or vocabulary (aOR 1.09), nor with verbal fluency (animal naming, adjustedexp (β)1.04), or pure processing speed (TMT-A, adjustedexp (β)0.97).

## 4 Summary and Discussion

In this pre-registered cross-sectional study, we replicated the key findings ofSchlemm et al. (2022)in an independent population-based sample of1651middle-aged to elderly participants of the Hamburg City Health Study.

First, we confirmed that the severity of cerebral small vessel disease is associated with the time spent in high-occupancy brain states, defined by functional MRI. More precisely, we showed that every 5.1-fold increase in the volume of supratentorial white matter hyperintensities of presumed vascular origin (WMH) was associated with a 0.95-fold reduction in the odds of occupying a brain state characterized by activation or suppression of the default-mode network, at any given time during the resting-state scan.

Second, we confirmed that the time spent in high-occupancy brain states at rest is associated with cognitive performance. More precisely, a 5%-reduction in the fractional occupancy of DMN-related brain states was associated with a1.02-fold increase in the time to complete part B of the trail making test (TMT).

In a pre-planned multiverse analysis, findings relating to our primary and, to a lesser extent, secondary hypotheses were robust with respect to variations in brain parcellations and confound regression strategies. Inconsistent results were found with the Desikan–Killiany parcellation, likely reflecting the notion that the spatial resolution and functional specificity of this coarse, structurally defined atlas are inadequate for analyzing functionally defined brain states. Across brain parcellations, effect sizes were smaller with the ICA-AROMA confound regression strategy and failed to reach nominal statistical significance. This might be due to a relatively large residual motion component in measures of dynamical functional connectivity after de-noising with ICA-AROMA, as described previously (Lydon-Staley et al., 2019).

We also confirmed across several brain parcellation resolutions that high-occupancy states at rest are characterized by either activation or suppression of the default mode network, reflecting its role as the predominant task-negative brain network.

In unplanned, exploratory analyses, we described the association between brain state dynamics and cognitive measures other than executive function and processing speed and reported a strong, preliminary association between time spent in high-occupancy states and delayed word recall.

We further explored, and report inAppendix Tables 1and2, the effect of motion; results relating to our primary and, to a lesser extent, secondary, hypotheses were robust to additional, unplanned adjustments for DVARS, RMSD, and mean framewise displacement.

The presented results provide robust evidence for a behaviorally relevant association between cerebral small vessel disease and functional brain network dedifferentiation.

Further research is required to replicate our findings in different populations, such as those affected more severely by cSVD or cognitive impairment, or being studied using different imaging protocols, to determine the generalizability of our findings with respect to varying operationalizations of the notions of cSVD, brain state, and cognition, and to understand the mechanisms underlying the reported associations.

## Figures and Tables

**Appendix Fig. 2.**
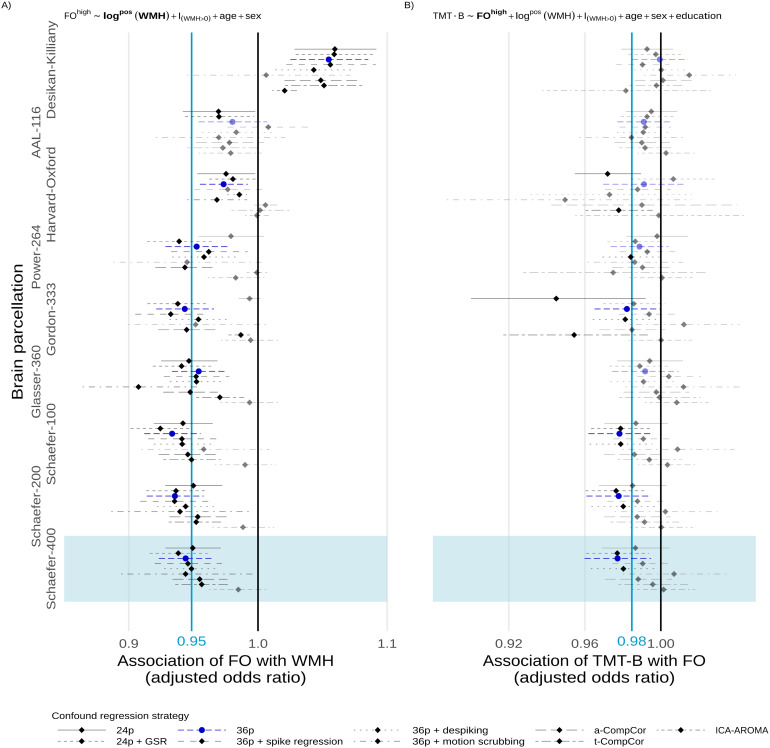
Multiverse analysis. Adjusted effect size estimates of the associations between cSVD severity (periventricular WMH volume) and network dedifferentiation (less time spent in high-occupancy DMN-related brain states) (A), and between network dedifferentiation and executive function (TMT-B completion time) (B). For details, see caption toFig. 5.

**Appendix Table 1.** Association between time spent in high-occupancy DMN-related brain states and WMH volume adjusted for age, sex, and motion parameters.

	Estimate	P	95%-CI
Intercept	0.32	<0.0001	0.28–0.36
WMH, per 5.1-fold increase ^1^	0.96	0.0004	0.94–0.98
Age, per 10 years	1.01	<0.0001	1.00–1.01
Female sex	1.11	<0.0001	1.08–1.15
$1_{\left\{\text{WMH}=0\right\}}$	0.91	0.3552	0.74–1.11
DVARS	0.98	<0.0001	0.98–0.99
RMSD	28.29	0.0055	2.67–299.8
Framewise displacement	0.16	0.0112	0.04–0.66

1 Interquartile ratio2.37/0.468=5.06.

**Appendix Table 2.** Association between TMT-B and time spent in high-occupancy DMN-related brain states adjusted for age, sex, WMH volume and years of education, and motion parameters.

	Estimate	P	95%-CI
Intercept	46.83	<0.0001	36.74–59.72
${\text{FO}}^{\text{high}}$ , per 5%	0.71	0.0718	0.49–1.03
WMH, per 5.1-fold increase ^1^	1.01	0.3414	0.98–1.04
Age, per 10 years	1.02	<0.0001	1.01–1.02
Female sex	1.00	0.8171	0.96–1.04
Education, per year	0.97	<0.0001	0.97–0.98
$1_{\left\{\text{WMH}=0\right\}}$	0.96	0.7581	0.73–1.29
DVARS	1.01	0.0001	1.00–1.01
RMSD	0.31	0.4695	0.01–7.45
Framewise displacement	1.08	0.9322	0.16–7.13

1 Interquartile ratio2.37/0.468=5.06.

## Data Availability

Preprocessed data and code are available athttps://github.com/csi-hamburg/HCHS-brain-states-RR, v2.1.1.https://doi.org/10.5281/zenodo.7337577.

## A1 Supplementary Results

### A1.1 Deep and periventricular WMH

Here we present, in analogy toFigure 5, the results of the multiverse analyses of the association between cSVD burden, FO of DMN-related states, and executive function, when cSVD is operationalized as the volume of deep or periventricular white matter hyperintensities, respectively.

### A1.2 Motion parameters

We also present, in analogy toTables 5and6, regression tables for the association between time spent in DMN-related brain states (FO) and WMH volume, and between TMT-B and FO, adjusted for DVARS, RSMD, and framewise displacement, in addition to age, sex, and, in the latter case, years of education.
